# Urban and individual correlates of subjective well-being in China: An application of gradient boosting decision trees

**DOI:** 10.3389/fpubh.2023.1090832

**Published:** 2023-05-19

**Authors:** Xiaoyan Huang, Chenchen Kang, Chun Yin, Yu Li

**Affiliations:** ^1^Northwest Land and Resources Research Center, Global Regional and Urban Research Institute, Shaanxi Normal University, Xi’an, China; ^2^School of Resource and Environmental Sciences, Wuhan University, Wuhan, China; ^3^International Institute of Spatial Lifecourse Health (ISLE), Wuhan University, Wuhan, China

**Keywords:** happiness, life satisfaction, built environment, urban environment, threshold effect, China, health city, machine learning

## Abstract

**Introduction:**

Subjective well-being (SWB) is attributable to both individual and environmental attributes. However, extant studies have paid little attention to the contribution of environmental attributes at the urban level to SWB or their nonlinear associations with SWB.

**Methods:**

This study applies a machine learning approach called gradient boosting decision trees (GBDTs) to the 2013 China Household Income Survey data to investigate the relative importance of urban and individual attributes to and their nonlinear associations with SWB.

**Results:**

The urban and individual attributes make similar relative contributions to SWB. Income and age are the most important predictors. Urban facilities make a larger contribution than urban development factors. Moreover, urban attributes exert nonlinear and threshold effects on SWB. Cultural facilities and green space have inverted U-shaped correlations with SWB. Educational facilities, medical facilities, and population size are monotonically associated with SWB and have specific thresholds.

**Discussion:**

Improving urban attributes is important to enhancing residents’ SWB.

## 1. Introduction

The pursuit of happiness is not only a basic demand of people but also the ultimate goal of human society (1, 2). On the one hand, a higher level of happiness reduces people’s negative emotions and protects against mental diseases (e.g., mental disorder and depression) (3, 4). On the other hand, a higher level of happiness promotes creativity and working efficiency, which helps to promote the development of the whole society (5). Hence, governments worldwide have aimed to enhance people’s subjective well-being (SWB) to promote both people’s quality of life and the overall development of society (6). For example, “ensuring healthy lives and promoting well-being for all at all ages” is one of the key sustainable development goals proposed by the United Nations (7). In the United Kingdom, the government proposed the inclusion of the well-being index in its General Household Survey. In China, promoting people’s SWB is an essential element of the Chinese Dream (8).

SWB is attributable to both individual internal endowments and the external living environment (9, 10), but there is little evidence suggesting which of these plays a more important role in promoting SWB. The majority of previous studies have suggested that individual sociodemographic characteristics are the most important determinants of SWB (11, 12). However, some scholars, particularly those in the field of urban planning, have argued that environmental attributes may play more important roles than individual sociodemographic characteristics in shaping SWB (13, 14) because sociodemographics (e.g., gender, age, and race) are difficult to intervene in. Hence, a comparison of the relative importance of urban and individual attributes to SWB can inform policymakers of the effectiveness of intervening in urban attributes in enhancing SWB. Moreover, by identifying the most important predictor of SWB, policymakers can design more targeted policies with which to promote SWB.

Previous studies on the associations between urban attributes and SWB have mainly been based on linear assumptions (15, 16), which can lead to biased results. A distinct example of this is that the same environmental element (e.g., green space) has been found to have mixed associations with SWB, including positive (17), negative (18), and non-significant associations, across studies (19). Recently, an increasing number of scholars have attempted to go beyond the linear assumption and explore the potential nonlinear and threshold associations of SWB with various predictors (11, 20). However, these studies have paid little attention to environmental attributes at the urban level and their nonlinear effects on SWB. It is necessary to identify the nonlinear associations between urban attributes and SWB to help policymakers reduce costs to obtain more benefits and promote greater precision in planning.

This study aims to answer two research questions. First, do urban attributes or individual sociodemographic attributes make a more important collective contribution to SWB? Second, do urban attributes have nonlinear associations with SWB? Based on the 2013 China Household Income Project (CHIP2013), this study uses a gradient boosting decision tree (GBDT) approach to investigate the relative contribution and nonlinear effects of urban attributes on SWB.

The remaining sections progress as follows. Section 2 is a review of the literature and that identifies the research gaps. Section 3 is a description of the data and methods. Section 4 presents the results. Section 5 offers a discussion of the main findings and policy implications. Section 6 presents the conclusion.

## 2. Literature review

Social-ecological theory posits that SWB is attributed to both individual and environmental attributes (21, 22). Many scholars believe that the majority of the variation in SWB derives from sociodemographic attributes (i.e., gender, age, and income) (12, 23) because SWB reflects the degree to which individual preferences are satisfied, which is mostly a measure applied at the individual scale (24). However, several recent studies have suggested that environmental elements also play a vital role in SWB (20, 25) as environmental elements are upstream factors of SWB that can affect it by influencing daily activities and moods (13). For example, using generalized structural equation models, Yin et al. (26) found that both environmental and sociodemographic attributes are important to life satisfaction. Moreover, they found that urban population density had a larger effect size than income, which was the most important sociodemographic attribute in their model. Yin et al. (27) also found that environmental attributes collectively contribute approximately 67% to the prediction of obesity, which was twice as high as the contribution of the collective sociodemographic attributes (31%). Considering that health and SWB are highly related to each other, this finding implies that environmental attributes may also be more important contributors to SWB than sociodemographic attributes. Since the current evidence on this topic is limited, policymakers wonder whether environmental attributes play a more important role in SWB.

Some studies have explored the association between environmental attributes and SWB and have found that walkable and green neighborhoods, as characterized by high density, mixed land use, connected streets, high levels of accessibility, and sufficient green space, have positive associations with SWB (28–31). However, these studies are based on environmental attributes at the neighborhood scale, which may not fully capture the environmental correlates of SWB, because people’s daily activities occur throughout the entire city rather than in a single neighborhood (32, 33). Several studies have suggested that urban attributes (e.g., urban development factors and urban facilities) play a more important role in shaping resident’s health and SWB (27, 34) than neighborhood attributes. On the one hand, people often travel from their neighborhoods to other neighborhoods for diverse reasons (e.g., working and shopping) (35). A study from Shanghai has suggested that both perceived residential and workplace environmental attributes can affect people’s SWB by influencing commuting satisfaction and place satisfaction (36). Moreover, people can also travel to other destinations aside from residential and workplace neighborhoods to hang out with friends, see doctors, and relax themselves. On the other hand, the positive and negative externalities resulting from urban attributes may also contribute to SWB by affecting people’s daily lives and moods (37, 38). For example, people have higher SWB when they are living in cities with higher accessibility (39), but their SWB tends to be lower in cities with higher levels of traffic congestion and air pollution (13, 37). Therefore, it is necessary to consider environmental attributes at the urban scale to identify the correlates of SWB.

Previous studies involving urban attributes only focus on the impact of singular urban attributes (e.g., a dummy variable indicating whether the resident lives in an urban area) on SWB. Few studies consider both urban development and urban facilities, which are two key dimensions of the urban attributes that relate to SWB. In terms of urban development factors, studies have found that urban economic development is positively related to SWB (40). Some studies have suggested that residents in large cities are happier than those in small and medium cities (41), but other studies have found that the higher that the population density is, the lower the SWB is (42–44). Few studies have explored the relationship between urban spatial structure and SWB. In theory, people living in a polycentric area with a good job-housing balance tend to be happier because they do not suffer from a long-distance commuting (45). Urban facilities are more directly related to people’s SWB because sufficient facilities help people meet their needs more easily. In particular, culture, education, and healthcare are conducive to promoting diverse, integrated, and positive urban interaction spaces, which can promote harmonious and equal urban social relationships, and enhancing the sense of urban identity and happiness (46). Green space has mixed relationships with SWB. Although green space and public transportation are important to SWB, previous studies have often focused on their associations with SWB at the neighborhood scale rather than that at the urban scale.

Moreover, previous studies have paid less attention to the nonlinear association between urban attributes and SWB. A well-known example is the happiness paradox, which suggests that the happiness level of the rich is higher than that of the poor, while economic development in a country does not necessarily improve happiness (47). The three-factor theory argues that the contributors to SWB can be classified into basic, performance, and excitement factors (48). Satisfactory basic factors are necessary to improving SWB, but basic factors make little contribution to SWB once the need for them are met. Excitement factors are not relevant to SWB at first, but they promote SWB rapidly once people become satisfied with them. Performance factors have linear associations with SWB (49). Based on this theory, some scholars have found that neighborhood environmental elements exert nonlinear and threshold effects on SWB (25, 50). However, the literature has paid little attention to the nonlinear and threshold effects of urban attributes on SWB.

**Figure 1:**
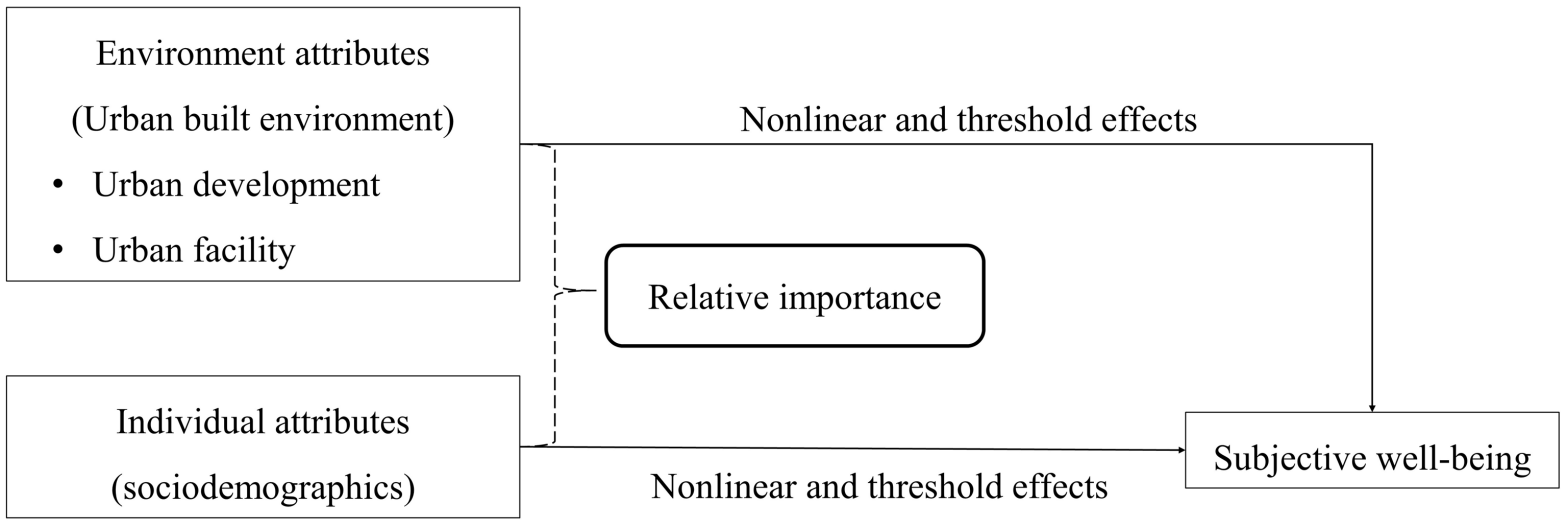
The empirical framework of this study.

Overall, we identify two major gaps in the literature. First, existing studies pay insufficient attention to the relationship between urban attributes and SWB, and it is still unknown whether urban attributes play a more important role in SWB than sociodemographics. Second, previous findings have not reached a consensus, and few studies have explored the manner in which urban attributes are nonlinearly associated with SWB. To address these gaps, we construct a theoretical framework to guide this study (Figure 1). In this framework, we assume that SWB is affected by both individual and environment attributes. We use individual sociodemographics and the urban built environment as proxies of individual and environment attributes, respectively. Moreover, we particularly focus on their relative contributions to and nonlinear associations with SWB, which may help scholars better understand the linkages and provide policymakers with insight into how to improve the urban environment to enhance SWB.

## 3. Materials and methods

### 3.1. Data

Data came from the China Household Income Project in 2013 (CHIP2013), administered by the China Institute of Income Distribution. CHIP2013 set an independent sampling frame and the data were collected in urban areas based on a stratified, multistage, and systematic sampling method. First, mainland China was classified into three regions, namely, the eastern, central, and western regions. Second, 126 cities across 15 provinces within those three regions were selected based on the number of households per city. Third, 7,935 urban households were selected within the selected cities. Further details can be found in Ma (51).

According to the literature (52, 53), a city refers to the city proper of a prefecture-level city. The aggregated urban data was primarily taken from the 2014 China City Statistical Yearbook and we merged them with the cities in which the respondents resided.

After merging the urban attributes and removing missing values, the working sample in this study comprised 5,736 respondents from 79 cities across 15 provinces. The 79 cities account for 27% of the prefecture-level cities in China and include 14 provincial capitals, accounting for 61% of the country’s provincial capitals.

### 3.2. Variables

SWB is the response in this study, which is measured with an overarching and single question, “All things considered, do you feel happy?,” with responses ranging from “very unhappy” (1) to “very happy” (5). This measure has often been used in the literature and has proven both effective and credible (54, 55).

The selected predictors include both urban attributes and sociodemographic characteristics. Urban attributes comprise urban development factors and urban facilities. Urban development factors include population size, population density, polycentricity index, the jobs-housing imbalance index, and the gross domestic product (GDP) *per capita*. Population size refers to the total resident population within the city proper (unit: 10,000 people). Population density is defined by population size divided by the area of the city proper in square kilometers. The polycentricity index refers to the balance between a city’s main employment center and its subcenters, and the jobs-housing imbalance index refers to the spatial mismatch between the number of jobs and the number of residential population. Both the polycentricity and jobs-housing imbalance indexes are measured following the method of Sun and Yin (33).

Urban facilities comprise bus, subway, greenness, cultural, educational, and medical services. In particular, the bus facility is measured by the number of buses per 10,000 population. The subway facility refers to the number of subway stations divided by the area of the city proper in square kilometers. Green space is measured by the area of green land and parks in square meters *per capita*. Cultural facilities are measured by the number of theaters, music halls, and cinemas per 10,000 population. Education facilities refer to the number of primary and secondary schools per 10,000 population. Medical facilities are calculated by the number of hospitals and health centers per 10,000 population.

Individual sociodemographic predictors include gender (male vs. female), age (in years), marital status (married/cohabitating vs. others), hukou status (local vs. migrant), level of education (as an ordered categorical variable), working hours per day (in hours), and annual income (in 10,000 yuan).

### 3.3. Method

To investigate the relationship between SWB and urban attributes, we used a machine learning technique known as gradient boosted decision trees (GBDTs). The GBDT method is derived from computer science and is used to predict data (56). Recently, urban planners have used this method to evaluate the nonlinear correlations of SWB with the built environment (11). Compared to traditional linear regression approaches, the use of GBDTs has certain strengths. First, it results in more accurate estimations because it accounts for the correlations between predictors (27) and is less vulnerable to potential outliers (57). Second, it allows for predictors with missing values and assumes that those missing values are due to a particular reason (27). Third, it can generate the relative importance of each predictor by measuring its potential to reduce prediction error relative to that of other predictors (58). Fourth, it can predict irregular nonlinear relationships between the predictor and the response by generating accumulated local effects plots (59). Moreover, compared to other machine learning approaches (e.g., XGBoost), GBDT often has better predictive power (60, 61).

The GBDT consists of a composite of decision trees and a gradient boosting algorithm. The decision tree approach divides the observations into various subsample regions and uses the mean of the response from each region to build the decision tree (62). The gradient boosting method combines multiple simple models into a complex model in a sequential manner and improves the model performance by identifying the best solution from a wide range of potential solutions (62). In particular, it predicts new residuals based on the residuals of the previous tree until the residuals can no longer be improved (25, 63).

We evaluate the GBDT model in R using the “gbm” package (64). Based on the literature (58, 65), we set the shrinkage of the model to 0.001, the depth of the tree to 10, and the maximum number of iterations to 8,000. A fivefold cross-validation process is applied to determine the optimal number of iterations and ensure the accuracy of the model (25). The GBDT model converges after 5,127 iterations, and the final value of *R*^2^ is 0.15.

## 4. Results

### 4.1. Characteristics of respondents and cities

Table 1 shows respondents’ characteristics and urban attributes. The SWB of respondents averages 3.71, which is between medium and happy. This is similar to the results of previous Chinese studies, which have shown an average Chinese SWB of 3.75 (66). Forty-eight percent of respondents are males with a mean age of 47 years old. Eighty-six percent of respondents are married, and 85% are local residents. The average level of education of respondents is 4.51, which represents a high school graduate. They report an average of 8 working hours per day, and their average annual income is approximately 40,000 yuan.

**Table 1:** Descriptive statistics of variables.

Variable	Mean/%	S.D.	Min	Max
Sociodemographic attribute				
Subjective well-being	3.71	0.80	1	5
Male	48%	—	—	—
Age	46.88	13.97	18	90
Married	86%	—	—	—
Local hukou	85%	—	—	—
Education	4.51	2.07	1	9
Working hours	8.03	2.32	0	18
Income	4.02	2.96	0	34.80
Urban attribute				
*Urban development*				
Population size	215.11	256.81	36.5	1783.1
Population density	1144.23	855.59	120.73	5119.80
Polycentricity index	0.31	0.29	0	1.50
Jobs-housing imbalance	0.07	0.05	0	0.24
GDP *per capita*	8.00	6.17	1.73	46.78
*Urban facility*				
Bus facility	9.70	11.06	1.45	98.53
Subway facility	0.09	0.24	0	0.88
Green space	10.29	7.20	3.24	59.34
Cultural facility	0.05	0.04	0.01	0.21
Educational facility	1.60	0.67	0.71	3.99
Medical facility	0.63	0.56	0.13	4.48

In terms of the urban attributes used in this study, the average population size is approximately 2.15 million people, and the mean population density is 1,144 people/km^2^. The mean polycentricity index is 0.31, and the jobs-housing imbalance index is 0.07. On average, the GDP *per capita* is 80,000 yuan. There are approximately 10 buses per 10,000 people, and the average density of subway stations is 0.09 unit/km^2^. The mean green space is approximately 10 m^2^ *per capita*. There are 0.05 cultural facilities, 1.6 schools, and 0.63 hospitals per 10,000 population in a city proper on average.

### 4.2. Relative importance

Table 2 presents the relative importance of each sociodemographic and urban attribute to the prediction of SWB. Their total relative contribution is 100%. Overall, sociodemographic attributes (50.5%) and urban attributes (49.5%) make similar collective contributions to the prediction of SWB, although the former makes a slightly larger one.

**Table 2:** Relative importance of predictors in the prediction of SWB.

Predictor	Relative importance	Rank
Sociodemographic attribute	Total 50.5%	
Income	14.9%	1
Age	13.6%	2
Married	6.7%	5
Education	5.8%	6
Working hours	5.6%	7
Male	3.4%	12
Local hukou	0.5%	18
Urban attribute	Total 49.5%	
*Urban development*	Total 19.2%	
Population density	4.9%	8
GDP *per capita*	4.0%	12
Population size	3.8%	13
Polycentricity index	3.6%	14
Jobs-housing imbalance	2.9%	16
*Urban facility*	Total 30.3%	
Cultural facility	9.8%	3
Educational facility	6.8%	4
Medical facility	4.4%	9
Bus facility	4.3%	10
Green space	4.2%	11
Subway facility	0.8%	17

Income is the most important predictor among all the predictors used. Its contribution accounted for 14.9% of the whole. Age is the second most important predictor, with a contribution of 13.6% in predicting SWB. Among sociodemographic attributes, marital status, education, working hours, and gender are also important predictors. Their relative contributions are 6.7, 5.8, 5.6, and 3.4%, respectively. Local hukou status exerts a negligible impact on SWB.

Among urban attributes, urban facilities play a more important role in predicting SWB than urban development factors (30.3% vs. 19.2%). Cultural services are the most important predictor among urban facilities, and they make the largest contribution among the urban attributes (9.8%). Education is the second most important predictor among urban facilities as well as among all urban attributes, with a relative importance of 6.8%. Among urban facilities, medical, green, and bus services make similar relative contributions of approximately 4%. The relative importance of subway service is negligible. A possible reason for this is that subways had not yet been built in most sample cities by 2013.

Urban development predictors show similar levels of importance in predicting SWB. Their relative contributions are between 2.9 and 4.9%. The most important urban development predictor is population density (4.9%), followed by GDP *per capita* (4.0%), population size (3.8%), and the polycentricity index (3.6%). The relative importance of the jobs-housing imbalance is small.

### 4.3. Associations between predictors and SWB

**Figure 2:**
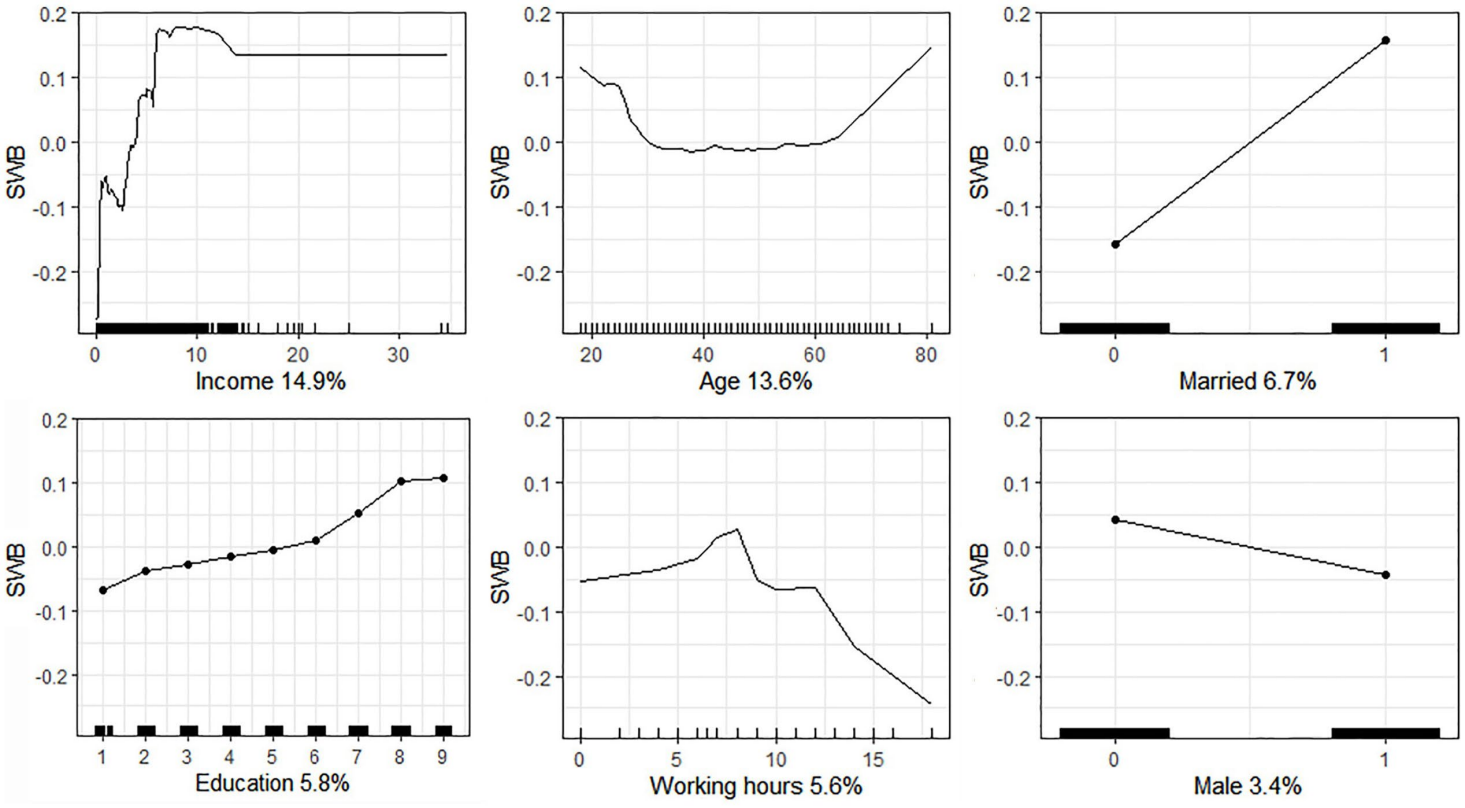
Associations between key sociodemographic attributes and SWB.

Figure 2 presents the association between key sociodemographic attributes and SWB using accumulated local effects (ALE) plots. In general, income has a positive association with SWB when it is below 140,000 yuan. Beyond this threshold, income makes no additional contribution to SWB. Age shows a U-shaped association with SWB. When the value of age is less than 30 years, it is negatively associated with SWB. When it is more than 60 years, however, it has a positive relationship with SWB. An age between 30 and 60 years makes a trivial contribution to SWB. Married respondents are more likely to have higher SWB. Education is positively related to SWB. In particular, an increase in the level of education from 6 (specialized secondary school) to 8 (undergraduate) is reflected in a more rapidly increasing SWB. Working hours show an inverted U-shaped association with SWB. When working hours are less than 8 h, longer working hours have a positive relationship with SWB. However, when working hours are more than greater than 8 h, longer working hours have a negative association with SWB, and the absolute value of its slope is much larger than that of the positive association. In general, males have lower levels of SWB than females in general.

**Figure 3:**
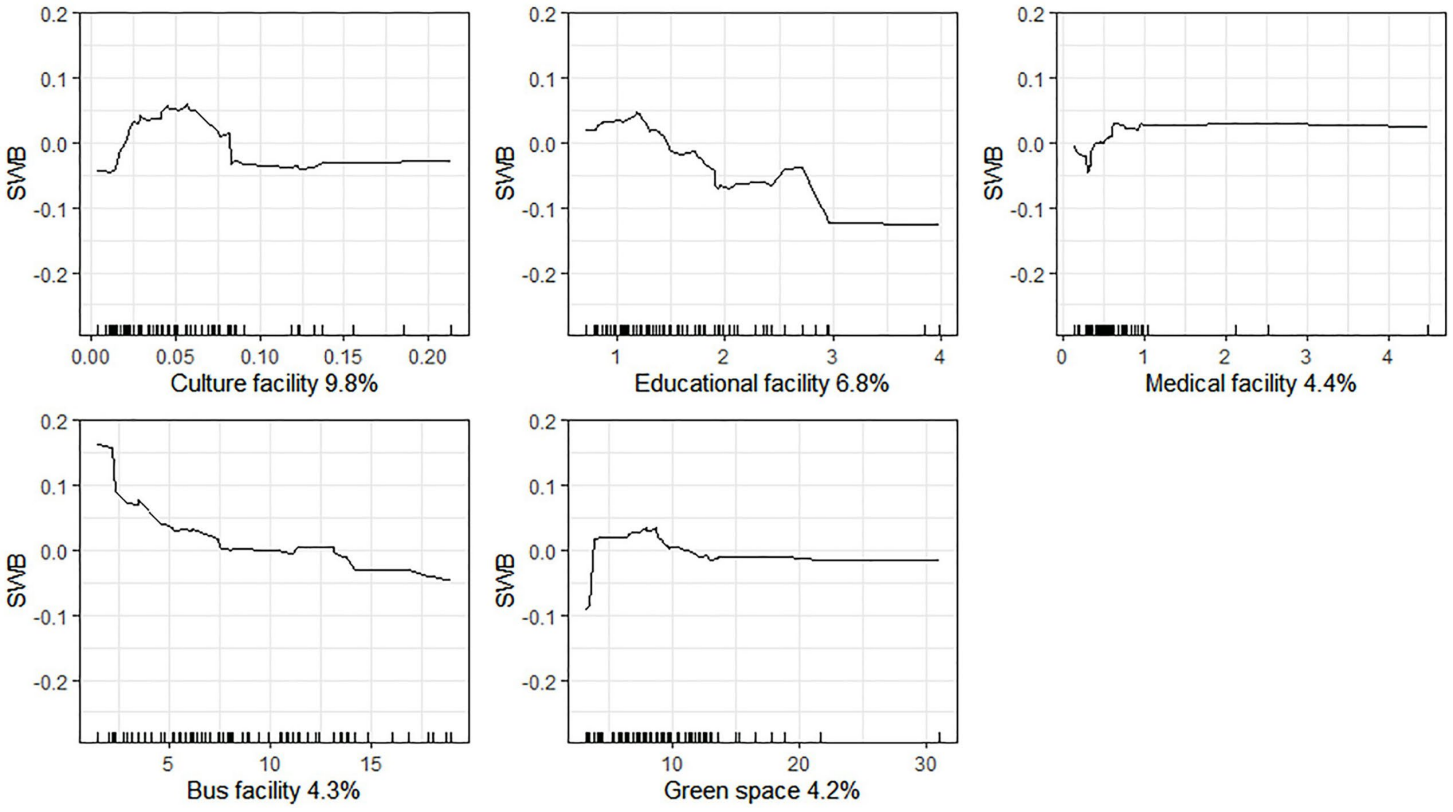
Associations between urban facility and SWB.

Figure 3 presents the associations between the predictors of key urban facilities and SWB. Cultural facilities show an inverted U-shaped association with SWB. In particular, an increase in cultural facilities from 0.01 to 0.06 per 10,000 population has a positive impact on SWB. However, when cultural facilities increase from 0.06 to 0.08 per 10,000 population, they become negatively related to SWB. Above a value of 0.08 cultural facilities per 10,000 population, cultural facilities make a small contribution to SWB. In general, education facilities have a negative relationship with SWB, while medical facilities have a positive association with SWB. The thresholds for these predictors are 3 schools per 10,000 population and 1 hospital per 10,000 population, respectively. Bus facilities are negatively associated with SWB. Compared to bus facilities with more than 7.5 vehicles per 10,000 population, those with fewer than 7.5 vehicles per 10,000 population have a larger negative slope when plotting their relationship with SWB. Green space also shows an inverted U-shaped association with SWB. When green space is below 9 m^2^/person, it has a positive relationship with SWB. However, green space between 9 and 14 m^2^/person is negatively associated with SWB. When green space is above 14 m^2^/person, this indicator makes no additional contribution.

**Figure 4:**
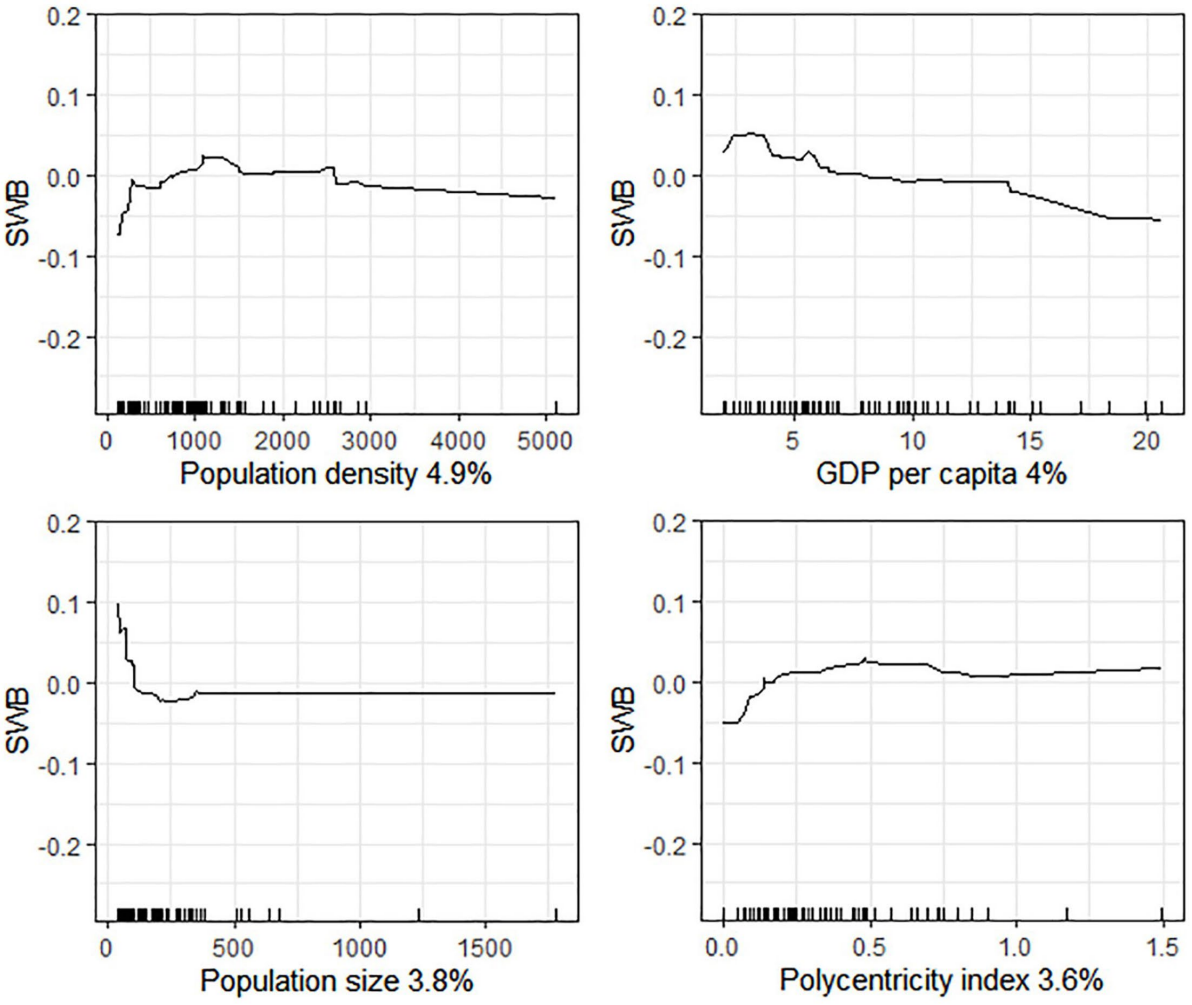
Associations between urban development and SWB.

Figure 4 presents the association between the key urban development predictors and SWB. The association between city population density and SWB is an inverted U-shaped curve. In particular, when the population density is below 1,200 people/km^2^, it has a positive association with SWB. However, beyond this threshold, it becomes negatively associated with SWB. GDP *per capita* show a negative association with SWB in general, which is in complete contrast to the association between household income and SWB. Population size is negatively associated with SWB, and its threshold is approximately 3 million people. Beyond this threshold, population size makes no additional contribution. The polycentricity index shows an inverted U-shaped association with SWB in general. When it is below 0.5, people in more polycentric cities usually report a greater SWB. However, when the polycentricity index is above 0.5, it has a negative association with SWB.

## 5. Discussion

Applying a GBDT approach to CHIP2013 data, this study explores the association of both urban elements and individual sociodemographic attributes with individual SWB in the Chinese context. This study has two main findings. First, both individual sociodemographics and urban attributes play important roles in predicting SWB. In particular, income and age are the most important predictors, and cultural and educational facilities are the most important urban attributes for the prediction of SWB. Second, most predictors have threshold and nonlinear associations with SWB.

Individual sociodemographics have an important impact on SWB. This is consistent with previous studies, which have found that individual sociodemographic attributes have significant impacts on SWB (67). Income and age are the most important indicators of SWB. Income is positively correlated with SWB in general. The economic level represents the material condition for the satisfaction of human needs, which is a fundamental factor for improving SWB (68). That is, a higher income improves people’s living standards and supports their daily needs, leading to a higher level of SWB (23, 69). However, when income exceeds the threshold, it ceases to exert any additional effects, which is in line with the World Happiness Report (70). According to Maslow’s theory of the hierarchy of needs, although a higher economic level tends to increase the SWB of people who are in a low-income stage, its effects may be replaced by those of higher-level needs (e.g., belongingness and self-actualization) once personal income have reached a certain level (71). The association between age and SWB plots as a U-shaped curve. Young adults and older adult people both exhibit higher levels of SWB than middle-aged people. This result is similar to the findings of a previous study, which showed that Chinese urban residents experience the lowest level of life satisfaction at 40 years of age (72).

In addition to individual sociodemographic attributes, urban attributes play an important role in SWB. This is aligned with social-ecological theory, which argues that SWB is attributable to both the environment and individual attributes (21). On the one hand, cities offer many activity destinations (e.g., hospitals, museums, and schools) that meet people’s diverse needs (73). On the other hand, cities have both positive (e.g., higher quality of education and health care) and negative (e.g., traffic congestion and air pollution) externalities, which are important contributors of SWB (37, 74). Moreover, urban facilities play a more important role than urban development elements. One possible reason for this is that urban facilities exert more direct effects on residents’ daily lives and their levels of SWB, while the effects of urban development factors are often indirect (20).

Urban attributes exert nonlinear and threshold effects on SWB. First, several urban attributes have complex nonlinear relationships, such as U-shaped and inverted U-shaped curves (e.g., cultural facilities, green space, and population density). Cultural facilities have an inverted U-shaped association with SWB. In particular, cultural facilities initially have a positive association with SWB. This is in line with previous studies, which have found that an increase in the number of cultural facilities is positively associated with both quality of life and life satisfaction (75, 76). A possible reason for this is that a greater number of urban cultural facilities (e.g., cinemas and theaters) enrich the cultural aspect of residents’ lives, resulting in a higher level of SWB (77). However, beyond the threshold, cultural facilities have a negative relationship with SWB. This is probably because when the number of cultural facilities *per capita* reaches a certain threshold, residents begin to pay more attention to their quality rather than their quantity. Studies have found that people have lower levels of SWB after accessing low-quality cultural facilities (78, 79). Another possible reason is that a continued increase in cultural facilities may cause several social problems, such as safety hazards, which have negative relationships with SWB (80).

Green space has an inverted U-shaped association with residents’ SWB. This attribute first shows a positive relationship with SWB, which is supported by existing studies (81, 82). A possible reason for this is that green space can improve residents’ health and can enhance the social cohesion and the sense of social identity, by offering space for activities, leisure, neighborhood communication, and contact with nature, thereby improving SWB (83). However, green space between 9 and 14 m^2^ *per capita* is negatively associated with SWB. This is probably because greater amounts of green space indicate that less space is dedicated to other types of land use, which affects destination accessibility and reduce life satisfaction (84). Moreover, in the Chinese context, the negative association may be a result of the imbalance between green park area and function, the weak functionality of city parks, and a lack of user-friendliness and recreational facilities.

The relationship between population density and SWB also followed an inverted U-shaped curve. Population density is positively associated with SWB at first. A higher population density implies more public services facilities that can promote residents’ accessibility (85) and social capital (86), which are important contributors to SWB. However, when population density exceeds 1,200 people/km^2^, we find that it becomes negatively related to SWB. This is in line with the literature (43, 87). A possible reason for this is that the higher population density in these ranges induces many negative externalities, such as congestion, noise, and insufficient public facilities *per capita*, thus lowering SWB (88).

Second, several urban attributes show a monotonic association with SWB with specific thresholds. Beyond the thresholds, these attributes do not exert additional effects (i.e., educational facilities, medical facilities, and population size). Educational facilities show a negative relationship with SWB. This finding is inconsistent with the literature, which has found that educational facilities have a positive association with SWB by enhancing the social sharing of resources (89). However, when schools are accessible to most urban residents, residents tend to pursue higher-quality schools. In the Chinese context, many residents have to endure higher rent and poorer housing quality to live in school catchment areas, which are often located in old town areas (90), thus leading to a lower SWB.

Medical facilities are positively related to SWB. This may be because medical facilities improve resources sharing among urban residents and increase their happiness (91). However, the effect of medical facilities on SWB has a threshold beyond which additional increases in the number of medical facilities do not result in additional SWB. Medical facilities are a basic factor according to the three-factor theory because when medical facilities are insufficient, they are positively related to SWB, but they have a limited effect on SWB once they exceed the threshold (49).

Population size is negatively correlated with residents’ SWB, which aligns with previous studies (92, 93). Citizens living in larger cities usually suffer from traffic congestion, an increased cost of living, and life pressure, which are harmful to SWB (83, 94). Further a continued expansion of the urban population drives up house prices and living costs, which is also harmful to SWB (95). Compared with the residents of megacities, the residents of small-and medium-sized cities often have higher levels of SWB due to their slower pace of life, more relaxed living environment, and lower living and housing costs (93).

Third, some factors presented monotonically increasing or decreasing influence (i.e., GDP *per capita*, bus facilities, and the polycentricity index). GDP has a negative association with SWB, which is supported by Easterlin’s paradox (47). Although China’s economy has grown continuously and rapidly, residents’ SWB has not correspondingly increased (96). This may indicate that both the minimum level at which people are satisfied and their happiness goals are increasing, leading to a decrease in overall happiness. In addition, the negative externalities generated in the process of economic growth have engendered problems such as resource mismatch, structural imbalance, environmental pollution, ecological damage, and lagging development of public services, thus weakening the positive impact on SWB (97).

Bus facilities are negatively related to SWB. This is consistent with the literature, which has found that people usually feel more uncomfortable on buses than in other travel modes (98–100). This is because bus services are often not punctual and uncomfortable (98, 101).

The polycentricity index has a positive relationship with the level of urban residents’ SWB, suggesting that living in polycentric urban areas tends to increase levels of SWB. The continued growth of urban polycentricity reduces agglomeration diseconomies and improves residents’ SWB by reducing transport costs and easing travel pressures (102). In addition, polycentricity reduces the monopoly of land located in the main center, thus potentially reducing the maximum land price and average land costs (103), improving the affordability of homeownership for residents, which in turn improves SWB.

These findings have some policy implications. First, policymakers should be confident that optimizing urban attributes is an effective way of enhancing people’s SWB because urban attributes collectively make a similar contribution to SWB as personal sociodemographic attributes do. Moreover, cultural and educational facilities need to make a higher priority when planning happier cities due to their greater relative importance. Second, policymakers need to pay more attention to thresholds in the relations of urban attributes to SWB because most urban attributes are nonlinearly related to SWB. In particular, when cultural facilities, green space, and population density are insufficient, policymakers should pay more attention to increasing their quantities to meet people’s basic needs, which helps enhance residents’ SWB. However, when their quantities are sufficient (i.e., reaching relevant thresholds), policymakers should focus to a greater degree on improving their qualities and promoting social equity to avoid reducing residents’ SWB through promoting competition for high-quality resources. Moreover, within certain thresholds, medical facilities are positively related to SWB. Therefore, overinvestment in medical facilities may not achieve additional benefits and may result in the waste of resources. However, educational facilities and population size are negatively associated with SWB within certain thresholds. Hence, policymakers need to realize that although equal educational facilities and larger city sizes are harmful to SWB, their negative effects will not increase once they reach their thresholds. Furthermore, GDP *per capita* and the polycentricity index are positively associated with SWB, and bus facilities are negatively related to SWB, but these associations do not have threshold effects. Therefore, to enhance people’s SWB, policymakers should continue investing in the promotion of urban economic development and adopting polycentric development strategies. Improving bus service quality rather than only focusing on its quantity may also benefit SWB. Third, income is the most important predictor of SWB, and it is positively related to SWB. Therefore, policymakers can improve residents’ SWB by increasing the minimum wage, which would be particularly important to the vulnerable population.

This study makes several contributions to the literature. First, the relative contributions of urban and individual sociodemographic attributes to SWB are explored in the study. We found a similar level of importance between urban attributes and sociodemographics, which highlights the fact that improving urban attributes plays an important role in the promotion of SWB. Second, this study elucidates the nonlinear associations between urban attributes and SWB by relaxing the linear assumption. The nonlinear and threshold effects help planners to design more accurate and efficient policies for intervening in urban environments. Third, it measures urban attributes across multiple dimensions rather than only measuring a singular variable (e.g., urban areas or not), which deepens the understanding of the association between urban attributes and SWB for both scholars and policymakers.

This study also has some limitations. First, because of data limitations, our study uses cross-sectional data and thus cannot identify causal relationships. Panel data should be used in the future to verify the effect of sociodemographic and urban attributes on SWB. Second, the GBDT approach reveals the relative importance of predictors, but it does not provide the statistical significance (*p* values). However, this is not a major issue, as the method is not used for statistical inference (104). Third, we do not identify the influential mechanisms of urban characteristics on SWB due to data unavailability. Future studies should explore the pathways of each urban attribute to SWB. Finally, this study is based on the Chinese context, which may not generalize well to other countries. In addition, the research data are relatively old and may not be suitable for the postpandemic era. Hence, we encourage more scholars to explore this association in different contexts and using alternate data to cross-validate our findings.

## 6. Conclusion

Using a GBDT model to the CHIP2013 dataset, this study explores the relative importance and nonlinear effects of urban attributes on SWB. The results show that urban and sociodemographic attributes have similar collective levels of importance (49.5% vs. 50.5%). The most important predictors are annual income and age. The relative importance of urban facilities (30.3%) is greater than that of urban development factors (19.2%). Cultural facilities, medical facilities and population density are the most important urban attributes in predicting SWB. Moreover, urban attributes have both nonlinear and threshold relationships with SWB. Cultural facilities and green space both have an inverted U-shaped relationship with SWB. Educational facilities, medical facilities, and city population size exert threshold effects on SWB. However, GDP *per capita*, bus facilities, and the polycentricity index have a linear association with SWB in general. Overall, urban attributes play important roles in SWB and exert nonlinear effects. Hence, to build happier cities, policy makers should pay more attention to the improvement of urban attributes.

## Data availability statement

The data analyzed in this study is subject to the following licenses/restrictions: Academic use only. Requests to access these datasets should be directed to Chinese Household Income Project, http://www.ciidbnu.org/chip/chips.asp?year=2013.

## Ethics statement

The studies involving human participants were reviewed and approved by the Institutional Review Board of China Institute of Income Distribution, Beijing Normal University. The patients/participants provided their written informed consent to participate in this study.

## Author contributions

XH: conceptualization, data curation, project administration, supervision, writing—original draft, and writing—review and editing. CK: conceptualization, data curation, formal analysis, software, visualization, writing—original draft, and writing—review and editing. CY: conceptualization, funding acquisition, methodology, project administration, software, validation, writing—original draft, and writing—review and editing. YL: formal analysis. All authors contributed to the article and approved the submitted version.

## Funding

This work was financially supported by the National Natural Science Foundation of China (Nos. 41831284, 41871168, and 42101184), the China Postdoctoral Science Foundation (Nos. 2020M681227 and 2022T150214), and the Funded Projects for the Academic Leaders and Academic Backbone, Shaanxi Normal University (No. 18QNGG013).

## Conflict of interest

The authors declare that the research was conducted in the absence of any commercial or financial relationships that could be construed as a potential conflict of interest.

## Publisher’s note

All claims expressed in this article are solely those of the authors and do not necessarily represent those of their affiliated organizations, or those of the publisher, the editors and the reviewers. Any product that may be evaluated in this article, or claim that may be made by its manufacturer, is not guaranteed or endorsed by the publisher.
